# In Defense of Metrics: Metrics Sufficiently Encode Typical Human Preferences Regarding Hydrological Model Performance

**DOI:** 10.1029/2022WR033918

**Published:** 2023-06-05

**Authors:** Martin Gauch, Frederik Kratzert, Oren Gilon, Hoshin Gupta, Juliane Mai, Grey Nearing, Bryan Tolson, Sepp Hochreiter, Daniel Klotz

**Affiliations:** ^1^ Institute for Machine Learning Johannes Kepler University Linz Austria; ^2^ Google Research Linz Austria; ^3^ Google Research Vienna Austria; ^4^ Google Research Tel Aviv Israel; ^5^ Department of Hydrology and Atmospheric Sciences University of Arizona AZ Tucson USA; ^6^ Department of Civil and Environmental Engineering University of Waterloo ON Waterloo Canada; ^7^ Google Research CA Mountain View USA

**Keywords:** hydrology, metrics, hydrograph, visual inspection, rainfall‐runoff, machine learning

## Abstract

Building accurate rainfall–runoff models is an integral part of hydrological science and practice. The variety of modeling goals and applications have led to a large suite of evaluation metrics for these models. Yet, hydrologists still put considerable trust into visual judgment, although it is unclear whether such judgment agrees or disagrees with existing quantitative metrics. In this study, we tasked 622 experts to compare and judge more than 14,000 pairs of hydrographs from 13 different models. Our results show that expert opinion broadly agrees with quantitative metrics and results in a clear preference for a Machine Learning model over traditional hydrological models. The expert opinions are, however, subject to significant amounts of inconsistency. Nevertheless, where experts agree, we can predict their opinion purely from quantitative metrics, which indicates that the metrics sufficiently encode human preferences in a small set of numbers. While there remains room for improvement of quantitative metrics, we suggest that the hydrologic community should reinforce their benchmarking efforts and put more trust in these metrics.

## Key Points

- A group of 622 participants visually judge model simulations similarly to quantitative metrics and considers a Machine Learning model best
- Nash–Sutcliffe efficiency and Kling–Gupta efficiency are good predictors of overall and high‐flow hydrograph quality but low‐flow metrics are poor predictors of low‐flow quality
- We can discriminate hydrographs that experts consistently consider good or bad purely based on existing quantitative metrics

## 1 Introduction

Decades of hydrological practice have produced an ever‐growing suite of metrics to quantify the performance of rainfall–runoff models. Nevertheless, as hydrologists, we often judge the quality of a model by looking at its hydrograph (Barthel et al., 2022). Despite continuous effort to push model evaluation to a less subjective basis (e.g., Alexandrov et al., 2011; Bennett et al., 2013), we lack an understanding of whether and how our intuitions are reflected in quantitative metrics (e.g., Crochemore et al., 2015; Mizukami et al., 2019; Schaefli & Gupta, 2007). Hence, in practice hydrologists sometimes distrust quantitative performance metrics as sole indicators of model skill (e.g., Houghton‐Carr, 1999; Legates & McCabe Jr., 1999; Moriasi et al., 2007; Pappenberger & Beven, 2004), and instead (or additionally) base modeling decisions on visual judgment (Chiew & McMahon, 1993; Rujner et al., 2018; Rykiel, 1996). This situation has two possible implications: either, (a) visual judgment substantially differs from metric‐based evaluation, and consequently the models that earlier benchmarks have reported as being “state of the art” may not actually be the best models, or (b) visual and metric‐based judgment are generally in agreement, and the sustained skepticism would be somewhat unjustified.

### 1.1 Overview

This study investigates and provides answers to three main research questions:

1. **Model ranking.** From the perspective of expert opinion, which hydrologic models seem to provide the most accurate hydrograph simulations? Further, do these expert‐based rankings agree with those derived using quantitative metrics?
2. **Metric ranking.** Which quantitative metrics are most informative of the expert opinion on hydrograph quality?
3. **Metric sufficiency.** Do existing quantitative metrics sufficiently‐well capture the desirable behavioral properties that experts look for in a hydrograph, or are there aspects to the visual assessment of hydrographs that are not suitably measured using existing quantitative metrics?

Note that the third question is slightly different from the one asked by Crochemore et al. (2015). While they found that no individual metric can fully replace expert judgment, we strive to analyze whether at least the full set of metrics, taken together, can do so.

We investigate these questions in a data‐driven way. In a large‐scale blind comparison study, we asked participants to compare pairs of unlabeled simulated hydrographs against streamflow observations, and to indicate which of the two simulated hydrographs they considered to be the better match to the data (compare: Crochemore et al., 2015). All of the simulated and observed data were drawn from a recent multi‐catchment hydrological model benchmarking study (Mai et al., 2022b) involving multiple physical‐conceptual‐based models (PC‐based; De la Fuente et al., 2021) and one machine‐learning‐based (ML‐based) model.

Notably, the responses of the blind‐comparison study paint a clear picture of model ranking (Section 2.3.1). The model simulations selected most often (by a significant margin) were those generated by the globally trained data‐driven Long Short‐Term Memory network approach (LSTM; Hochreiter & Schmidhuber, 1997). Next highly ranked were simulations generated by traditional PC‐based models that were calibrated per basin (i.e., locally). Traditional PC‐based models that were calibrated using regional schemes received the worst ratings. Overall, these results largely coincide with the KGE‐based rankings resulting from the original model intercomparison study (Mai et al., 2022b).

As one might expect, the quantitative metrics that were most indicative of the above‐mentioned rating outcomes changed when the participants were asked to focus on specific parts of the hydrographs, such as high or low flows (Section 2.3.3). Our results reveal that KGE, NSE, and Pearson's correlation are good predictors of participant ratings when the focus was directed to high flows and to overall hydrograph behavior. Surprisingly, however, when the focus was directed to low‐flow behavior, conventional dedicated low‐flow metrics proved to be remarkably uninformative. This highlights the considerable room for improvement with regards to such metrics.

The findings regarding metric sufficiency are double‐edged. On the one hand, we show that “visual” judgment is indeed subject to noise and inconsistency (Section 3.3). On the other hand, we also show that the subset of intersubjective (i.e., agreed upon) visual judgments can be explained and quantified using existing metrics. Moreover, our results indicate that we can discriminate “good” from “bad” hydrographs purely based on metrics (Section 3.3).

In summary, these results lead us to suggest that the hydrological community can trust the “hard numbers” from quantitative metrics of benchmarking efforts (if multiple metrics are used), and ground their modeling decisions on them.

### 1.2 Related Work

The large number of available hydrologic models raises the obvious challenge of how one should evaluate and compare models when faced with the need to choose the most suitable model for a given task (e.g., Garcia et al., 2017; Gupta et al., 2009; Krause et al., 2005; Moriasi et al., 2015). Benchmark studies are one approach that can help address this problem, and thus have gained substantial traction (e.g., Arsenault et al., 2022; Best et al., 2015; Gauch et al., 2021; Koch & Schneider, 2022; Kratzert et al., 2019; Lees et al., 2021; Mai et al., 2022b). Overall, the community usually bases their strategy for model performance evaluation and comparison on sets of quantitative metrics. Most commonly, these metrics include the ubiquitous Nash–Sutcliffe Efficiency (Nash & Sutcliffe, 1970) and the more recent Kling–Gupta Efficiency (Gupta et al., 2009).

At the same time, hydrologists continue to raise caution about the dangers of overreliance on quantitative metrics (e.g., Moriasi et al., 2007; Pappenberger & Beven, 2004), pointing to visual inspection of the raw hydrographs as an important strategy for model evaluation (Bennett et al., 2013; Moriasi et al., 2015; Rujner et al., 2018; Rykiel, 1996; Van Liew et al., 2005). Accordingly, the community has attempted to mold the visual evaluation process into a formal procedure (e.g., Barthel et al., 2022; Boyle et al., 2000; Pappenberger & Beven, 2004; Reusser et al., 2009; Wagener et al., 2003).

Arguably, these efforts have been of limited success, as evidenced by studies that have examined the relationship between results obtained via quantitative metrics and those based on visual assessment: most recently, Crochemore et al. (2015) surveyed 150 hydrologists and reported that visual judgment often yields results that cannot be represented by a single quantitative metric. Similarly, Houghton‐Carr (1999) found substantial disagreement between two judges in a smaller study. An earlier example by Chiew and McMahon (1993) investigated the relation between visual and quantitative evaluation by asking 63 experts to classify simulations into categories between perfect and unusable, based on both visual and numerical criteria. Their participants reported that they found visual indicators to be more important than the numerical ones. Overall, the authors reported that they were able to derive some basic patterns of numerical criteria that indicate good simulations, whereas the metrics were less useful for pinning down poor simulations.

One drawback of these previous studies comparing expert judgment with quantitative metrics is that their low numbers of participants may limit the reliability and representativeness of their results. While our study cannot guarantee these properties, it enables us to draw conclusions from a much larger pool of data, because we collected more than 14,000 ratings from over 600 participants.

We also note that the phenomenon of inconsistent expert decisions is not limited to hydrology. Rather, such disagreements are well‐known in social sciences. Researchers have found them in a variety of disciplines ranging from medicine to law, both among decisions of different experts and decisions of a single expert (e.g., Brown, 1983; Danziger et al., 2011; Hoffman et al., 1968; Shanteau, 1988). The style of graphical presentation may also play a role in the outcome of visual judgment. In fact, the effect of different visual presentations on human understanding and judgment has been the subject of a large body of research in the graphical perception community (e.g., Carpenter & Shah, 1998; Ratwani et al., 2008; Shah & Freedman, 2011). Researchers suggest a complex interplay between the recipient's experience in reading the chosen type of graph, their expertise in the domain of the depicted data, and the conclusions drawn from the display (Shah & Freedman, 2011). In our study, we focus on the centuries‐old technique of line charts to visualize time series (Playfair, 1786). Specifically, we use time as the *x*‐axis and discharge as the *y*‐axis, which may be one of the most common types of hydrologic prediction visualization (e.g., Beven, 2011). We show the simulated and observed hydrographs as lines in a single plot, which Javed et al. (2010) suggest are well suited for comparisons of few, short time series with limited overlap.

## 2 Methods

### 2.1 Study Design

**Figure 1:**
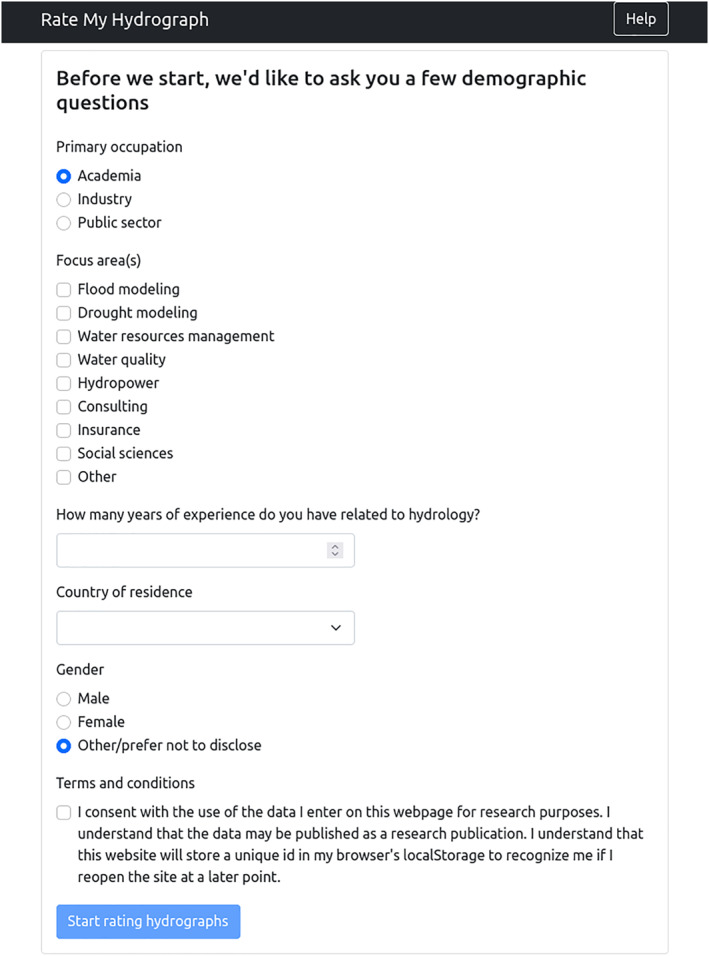
Screenshot of the questionnaire.

**Figure 2:**
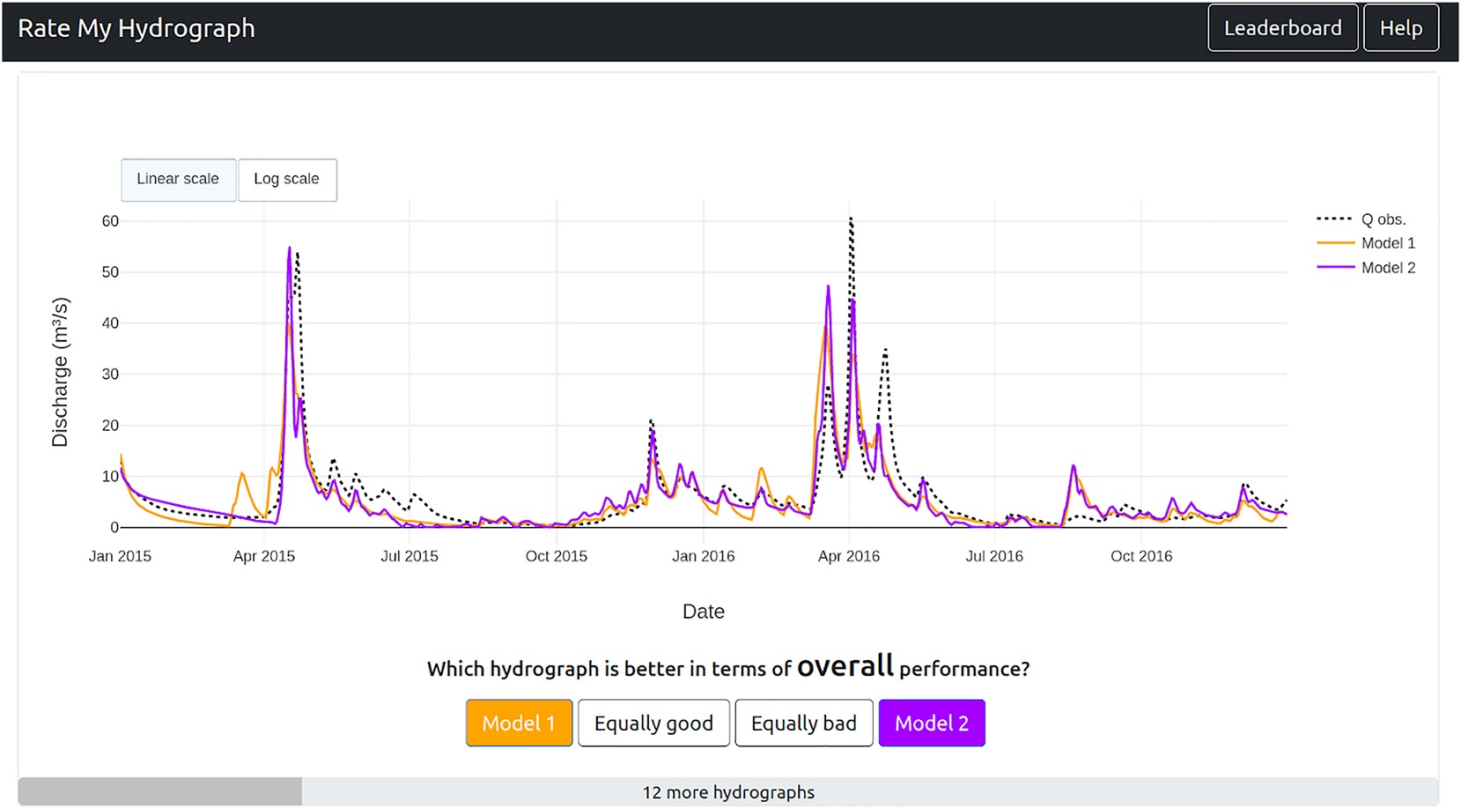
Screenshot of the rating page, showing two simulated hydrographs (solid lines) and the corresponding observed hydrograph (dotted). Participants could give their rating via the buttons at the bottom of the page (model 1 is better, model 2 is better, equally good, or equally bad). Further, there exists an option to switch between a linear and a logarithmic y scale.

The study was conducted via an openly accessible website where participants were first required to fill out a questionnaire consisting of demographic questions (see Figure 1). Participants were then asked to compare the simulations of two randomly selected models with the corresponding observed hydrograph on a randomly selected 2‐year period. To hide the names of the underlying models, the simulations were labeled *Model 1* and *Model 2*, while the observations were labeled *Q obs*. The participants had to select from one of four options: (a) model 1 better matches the observations, (b) model 2 better matches the observations, (c) both models are equally good, and (d) both models are equally bad (see Figure 2). Participants could proceed to rate as many sets of hydrographs as they liked; we recommended that they rate at least 15. For every 5 comparisons in a row, we asked participants to direct the focus of their comparisons on either (a) overall flows, (b) high flows, or (c) low flows. All hydrograph data (simulations and observations) were drawn from the Great Lakes Runoff Intercomparison Project (GRIP‐GL; Mai et al., 2022b), wherein experts calibrated their individual models and contributed simulations to the intercomparison study. Section 2.2 provides more detail on the GRIP‐GL study and the data we used from that study.

We solicited participants mainly via Twitter and email, but also through an oral presentation at the EGU General Assembly 2022 (Gauch et al., 2022).

The study was conducted in two rounds. In the first round (22 March to 6 July 2022), we collected ratings for random pairings of models for all basins. In an additional second round (6–20 July 2022), we restricted the possible comparisons to a smaller subset of hydrographs to collect a larger number of duplicate ratings for this subset. This allowed us to assess the consistency of ratings.

### 2.2 Data Source

All data used in our study originate from the Great Lakes Runoff Intercomparison Project Phase 4 (GRIP‐GL; Mai et al., 2022b), where experts contributed their best‐effort hydrograph simulations from their preferred models to a blind (i.e., post‐hoc) evaluation. To ensure a rigorous and fair benchmark, the experts calibrated their models of choice using a common training period (2000–2010, called calibration period in Mai et al., 2022b) for two sets of basins from the Great Lakes region in the USA and Canada. The first set of basins, called “objective 1,” comprised 66 basins with low human impact, while the second set, “objective 2,” comprised 104 basins that directly drain into one of the Great Lakes or the Ottawa River. This latter set of (most‐downstream) basins partly overlaps with the set of low‐impact basins from objective 1, but also includes basins that have been subjected to human influence. Following the practice of blind evaluation, participants had no access to data from the final held‐out test period (2011–2017, called validation period in Mai et al., 2022b) while they trained (calibrated) their models. The 13 models contributed to the study are listed in Table 1; they include a Deep Learning model, PC‐based models calibrated on a per‐basin basis, and PC‐based models calibrated on a regional basis. Using model predictions generated by these calibrated models on the held‐out test period, their runoff simulations were evaluated in terms of the KGE metric for gauged and ungauged settings. Models that also generate simulations of actual evapotranspiration, surface soil moisture, and snow–water equivalent were further evaluated on those values.

**Table 1:** List of Participating Models in the Great Lakes Runoff Intercomparison Project Phase 4

Model name	Lead modeler(s)	Routing scheme	Temporal resolution	Spatial resolution
Machine Learning model(s) (global calibration)				
LSTM‐lumped	Gauch, Klotz & Kratzert	None	Daily	Basins (141 + 71)
Hydrologic and land‐surface model(s) with calibration of each gauge individually (local calibration)				
LBRM‐CC‐lumped	Waddell & Fry	None	Daily	Basins (141 + 71)
HYMOD2‐lumped	Rasiya Koya & Roy	None	Daily	Basins (141 + 71)
GR4J‐lumped	Mai & Craig	None	Daily	Basins (141 + 71)
HMETS‐lumped	Mai & Craig	None	Daily	Basins (141 + 71)
Blended‐lumped	Mai, Craig & Tolson	None	Daily	Basins (141 + 71)
Blended‐Raven	Mai, Craig & Tolson	Raven	Daily	LSS–subbasins (2,187 + 2,170)
				Rout.–subbasins (2,187 + 2,170)
VIC‐Raven	Shen & Tolson	Raven	LSS–6 hr	LSS–grid (10 km)
			Rout.–daily	Rout.–subbasins (2,187 + 2,170)
Hydrologic and land‐surface model(s) with calibration of entire regions (regional calibration)				
SWAT‐Raven	Shrestha & Seglenieks	Raven	Daily	LSS–subbasins (3,230 + 2,268)
				Rout.–subbasins (2,187 + 2,170)
WATFLOOD‐Raven	Shrestha & Seglenieks	Raven	Hourly	LSS–grid (10 km)
				Rout.–subbasins (2,187 + 2,170)
MESH‐CLASS‐Raven	Temgoua & Princz	Raven	LSS–30 min	LSS–grid (10 km)
			Rout.–daily	Rout.–subbasins (2,187 + 2,170)
MESH‐SVS‐Raven	Gaborit & Princz	Raven	LSS–10 min	LSS–grid (10 km)
			Rout.–6 hr	Rout.–subbasins (2,187 + 2,170)
GEM‐Hydro‐Watroute	Gaborit	Watroute	LSS–10 min	LSS–grid (10 km)
			Rout.–hourly	Rout.–grid (1 km)

*Note*. The table lists the participating models and the lead modelers responsible for model setups, calibration, and validation runs. The models are separated into three groups (see headings in the table), namely a machine learning (ML) model which is globally calibrated, hydrologic models that are calibrated at each gauge (local calibration), and models that are trained for each region, such as the Lake Erie or Lake Ontario watershed (regional calibration). Note that the temporal and spatial resolution of the fluxes of the land surface scheme (LSS) can be different from the resolutions used in the routing (Rout.) component. All LSS grids are set to the RDRS‐v2 meteorological data forcing grid of around 10 by 10 km. The two numbers given in the column specifying the spatial resolution (X + Y) correspond to the spatial resolution of the models regarding calibration basins (X) and validation basins (Y). Source: Mai et al. (2022b).

For our study, we focused on the gauged GRIP‐GL streamflow results from both objectives (low human impact and most downstream). We did not use ungauged results, since these would have introduced an additional degree of freedom and, further, would have included a non‐negligible fraction of very poor simulations. The 13 models, 7 test period years, 141 basins from both GRIP‐GL objectives, and 3 rating tasks (overall, high flow, low flow) lead to a total of 198,900 possible rating situations, out of which we randomly drew the samples that were shown to participants.

### 2.3 Analyses of Results

#### 2.3.1 Model Ranking

As the ratings consist of pairwise model comparisons, we can derive a model ranking from the “win percentage,” that is, the number of times a model “won” in comparison with other models, divided by the overall number of won and lost comparisons of this model. The model for which this measure is highest (lowest) is the one that participants most often considered to be superior (inferior).

#### 2.3.2 Rating Consistency

The consistency of responses is important to the interpretation of our results. We quantify the quality of responses using data from the second part of the study, where we collected ratings for a smaller subset of 870 settings to generate a larger number of duplicates. We constructed this subset as those settings that were part of an existing “triangle”: for any set of models {*A*, *B*, *C*} and any specific basin, date range, and rating task, there exists one rating for the comparison model *A* versus model *B*, one for model *B* versus *C*, and one for model *C* versus *A* (Table 2 shows two examples). To ensure that we obtain sufficient duplicate ratings, we showed settings from 10 randomly selected triangles more often (in 1/3 of the ratings that were shown to participants) than the remaining ones.

**Table 2:** Examples for Two Triangles of Ratings for Three Models

Model A	Model B	Basin	Date range	Rating task	Rating
SWAT	GR4J	012345	2008–2009	Overall	Model *B* better
GR4J	LSTM	012345	2008–2009	Overall	Model *B* better
LSTM	GR4J	012345	2008–2009	Overall	Model *A* better
MESH‐SVS	VIC	123456	2008–2009	High flow	Model *B* better
VIC	HYMOD2	123456	2008–2009	High flow	Model *B* better
HYMOD2	MESH‐SVS	123456	2008–2009	High flow	Model *B* better

*Note*. The triangle formed by the first three ratings is consistent, as the ratings are not conflicting. The triangle formed by the second set of three ratings is inconsistent, as transitivity based on the first two ratings would suggest that HYMOD2 is better than MESH‐SVS.

We focus on two aspects of consistency: first, the agreement of multiple ratings for the same setting, and second, the consistency across related ratings.

Where we have collected multiple ratings of the same setting (same models, basin, date range, and task), we calculate the consistency of these ratings (a) on the basis of individual raters, and (b) on the basis of a hypothetical “expert panel.”

(a) **Individual raters.** We measure the agreement of the different ratings. To do so, we frame the ratings as outcomes of a classification task: we view each participant's rating as a “prediction” of what they believe to be the correct judgment. Given multiple such predictions for the same setting, we arbitrarily choose one of them as the “correct” one and calculate how well the remaining ratings agree with it. As a metric of this agreement, we use the standard tool set of classification evaluation: accuracy, precision, recall, and F1 scores (see Appendix B for the definition of these metrics). Since we do not know which participant's rating is correct, we repeat this leave‐one‐out process for all ratings until every rating acted as the correct one once. Finally, we average the metrics across these repetitions. As an example, if there exist three ratings of the same setting, we derive each metric (accuracy, precision, recall, F1 score) as the average of three values: each of these values is the respective metric calculated on all but one rating when we consider the remaining rating the correct one. Hence, if all ratings agree, this would result in perfect classification metrics: no matter which rating we consider correct, the remaining ones will agree.
(b) **Expert panel.** We define the “correct” rating as the most‐agreed class among all but one ratings (we randomly pick one of the classes if the majority vote is tied). This gives us a single classification outcome (which compares the remaining rating with the majority vote). We repeat this leave‐one‐out procedure until each rating was held out once, and calculate the classification metrics on the resulting set of rating–majority pairs.

In an additional analysis, we consider “triangles” of comparisons between three models in the same setting (same basin, date range, and task). With three possible answers for each rating (model *A* better, model *B* better, equal; note: to keep the consistency analysis of triangles simple, we group the two answers “equally good” and “equally bad” into one “equal” category), each rating triangle can have 27 possible outcomes. Out of these 27 outcomes, we consider 13 outcomes as consistent and the remaining 14 outcomes as inconsistent (inconsistent outcomes are: circles, where model *A* > *B*, *B* > *C*, *C* > *A*; double equalities, where model *A* = *B*, *B* = *C*, but *A* < > *C*; single equalities, where *A* = *B*, *B* > *C*, *C* > *A*). While one might argue about the (in‐)consistency of some of these cases—especially those that involve equalities—, we consider this definition of consistency an intuitive and simple heuristic to get a rough estimate of the rating quality. As a baseline, if participants rated entirely at random, the fraction of consistent ratings would be 13/27 ≈ 48.15%. If visual judgment captures some underlying notion of goodness of fit, we would expect our results to be of considerably higher consistency than this baseline.

#### 2.3.3 Metric Ranking

To determine which quantitative metrics best reflect the preferences of the experts, we performed a post‐hoc analysis of the collected ratings: we trained a random forest classifier to predict the ratings based on the quantitative values of the metrics computed for the two simulations that were rated. This means that the metrics are our explanatory variables and the expert opinions are the response variable (classification labels). Random forests (RF; Breiman, 2001) are well suited for this application, as their decision tree structure makes them highly interpretable and provides a quantitative measure of feature importance (Breiman et al., 1984), which indicates how much any individual input feature (in our case, a metric) contributed to the predicted classification. Features (metrics) that are assigned high importance are those that best explain (i.e., capture the most information about) the actual rating outcome. Table 3 provides a list of the metrics we used as input features in this assessment.

**Table 3:** Evaluation Metrics Used in This Study

Metric	Description	Reference
NSE	Nash–Sutcliffe efficiency	Equation 3 in Nash and Sutcliffe (1970)
logNSE	Nash–Sutcliffe efficiency in logarithmic space	
MSE	Mean squared error	
RMSE	Root mean squared error	
KGE	Kling–Gupta efficiency	Equation 9 in Gupta et al. (2009)
logKGE	Kling–Gupta efficiency in logarithmic space	
Pearson *r*	Pearson correlation between observed and simulated flow	Pearson (1895)
*α*‐NSE	Ratio of standard deviations of observed and simulated flow	From Equation 4 in Gupta et al. (2009)
*β*‐NSE	Difference of mean simulated and observed flow, divided by the standard deviation of observations	From Equation 10 in Gupta et al. (2009)
*β*‐KGE	Ratio of mean simulated and mean observed flow	Gupta et al. (2009)
FHV	Top 2% peak flow bias	Equation A3 in Yilmaz et al. (2008)
FLV	Bottom 30% low flow bias	Equation A4 in Yilmaz et al. (2008)
FMS	Bias of the slope of the flow duration curve between the 20% and 80% percentile	Equation A2 Yilmaz et al. (2008)
Peak timing	Mean time lag between observed and simulated peaks	Appendix A in Gauch et al. (2021)

#### 2.3.4 Metric Sufficiency

Quantitative metrics promise a principled assessment that allows to quantify the quality of a hydrograph. However, despite the existence of a wide array of hydrologic metrics, we still have little understanding of how well these metrics reflect the preferences of experts. To investigate whether there are patterns in expert ratings that are not captured by existing metrics, we train another RF‐based model to predict ratings from the metrics computed for the corresponding models.

In addition to that model, we train a further model that predicts expert ratings directly from the observed and the two simulated hydrographs—in other words, from the same data that experts had access to when they submitted their ratings. Since the hydrographs are time series, we use the Gated Recurrent Units architecture (GRU; Cho et al., 2014) that is designed to process time series (we also tested LSTMs, RNNs, and CNNs with similar but slightly worse results). GRUs are a type of recurrent neural networks that are similar to LSTMs and have fewer parameters. Unlike in the metric ranking analysis, here we train a single model on all rating tasks and provide the models with a flag that indicates the task. Specifically, to achieve robust estimates of the model performance, we train and evaluate the GRU‐based model and the aforementioned RF‐based model in a five‐fold cross‐validation setting. That is, we partition the data into five groups, train/validate on four of them and test on the remaining one (using nomenclature adopted from the ML convention). We repeat this process five times, such that we eventually test on each sample once, and average the classification results across these repetitions.

Importantly, the difference in prediction accuracy between the RF‐based (based purely on metrics) and the GRU‐based (based purely on raw hydrographs) models indicates whether the hydrographs contain information about expert ratings that the metrics are unable to provide. Since all metrics can be computed from the hydrographs, the GRU‐based model has access to at least as much information as the RF‐based one and should therefore be at least as accurate (minor differences in accuracy may be explained by the fact that the GRU has to learn more complex calculations based on the same amount of data).

Therefore, we posit that: If the GRU‐based model achieves considerably higher accuracy than the RF‐based one, this indicates that there exist patterns in the raw data that are informative of the expert ratings but are not captured by the tested suite of metrics. If, on the other hand, the GRU‐based model is no better than the RF‐based one (and if both models are significantly better than random chance) this indicates that the suite of tested metrics does already reflect the preferences that experts consistently express when rating hydrographs.

## 3 Results and Discussion

### 3.1 Participation

**Figure 3:**
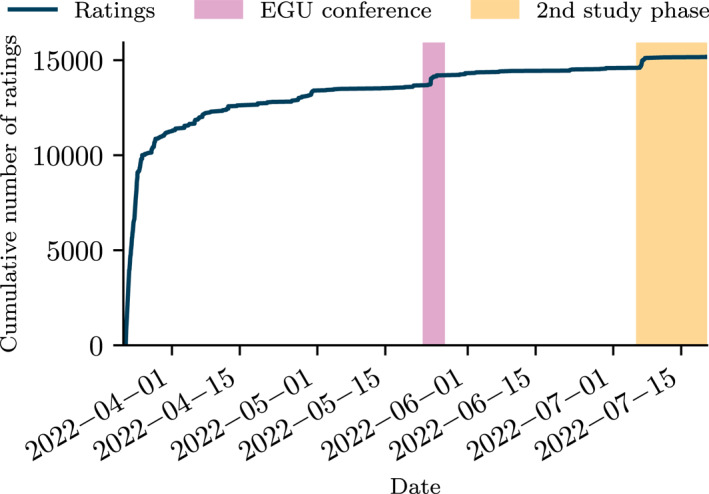
Cumulative number of ratings over time.

**Figure 4:**
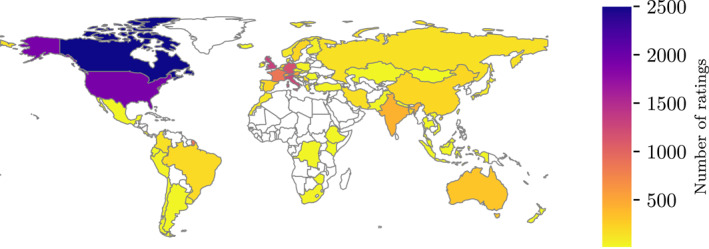
Map of the number of ratings by country.

Over the course of approximately 4 months between March and July 2022, 622 participants filled out the questionnaire and rated a total of 14,586 hydrographs (the main phase of the study). In the second study phase, 32 users provided an additional 589 ratings. Figure 3 shows the accumulation of ratings over time. Participants were from all over the world, predominantly from North America and Europe, with clear underrepresentation of African countries (see map in Figure 4). The spatial distributions of participants and ratings were similar, as the number of ratings per participant was relatively stable across space.

**Figure 5:**
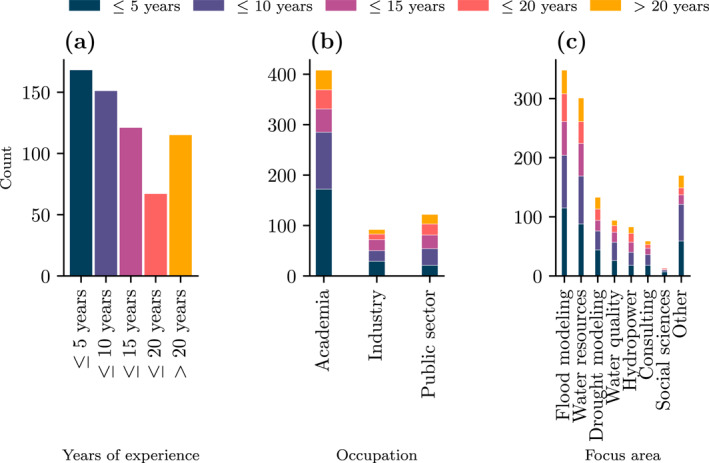
(a) Histogram of participants' experience, (b) histogram of participants' occupation, and (c) most common focus areas.

Most of the participants (408; 66%) were from academia, especially those with fewer years of experience (see overall distribution of experience in Figure 5a). Still, a significant portion were from industry (92; 15%) and the public sector (122; 20%). The most common focus area reported by participants was flood modeling, followed by water resources and drought management (Figure 5c).

### 3.2 Model Ranking

A first result we can directly draw from the ratings is a ranking of models. Whereas benchmark studies in hydrology commonly rank models by their results according to one or multiple metric(s) (e.g., Best et al., 2015; Kratzert et al., 2019; Mai et al., 2022b), we rank models according to the number of times a model “won” in direct comparison with another model.

Table 4 shows that the LSTM‐based model clearly dominates the other models in the ratings. Across all rating tasks (i.e., overall, high flow, and low flow combined), it has a win percentage of 86%, which is significantly higher than that of the next‐best models (GR4J and the Blended‐lumped model, with win percentages of 67% and 64% across all rating tasks, respectively). This pattern holds across all rating tasks (overall, high flow, low flow). However, it is most pronounced for low‐flow ratings (LSTM win percentage 90%) and least pronounced for high‐flow ratings (LSTM win percentage 81%). In fact, all of the best ranked models have lower win percentages in the high‐flow ratings. Moreover, the LSTM‐based model was least likely to be considered “equally bad”—only 11% of the LSTM ratings were “equally bad,” whereas all other models were given this rating at least 19% of the time.

**Table 4:** Win Percentage and Median Validation Period Kling–Gupta Efficiency From the Great Lakes Runoff Intercomparison Project Phase 4 by Model for the Different Rating Tasks

	Rating task	Win percentage			Median KGE from GRIP‐GL
		Overall	High flow	Low flow	
Regionally calibrated	mesh‐class‐raven	22	33	18	0.45
	gem‐hydro‐watroute	23	22	35	0.46
	mesh‐svs‐raven	32	32	50	0.57
	swat‐raven	33	32	35	0.56
	watflood‐raven	36	37	33	0.62
Locally calibrated	lbrm‐cc‐lumped	49	53	42	0.75
	hymod2‐lumped	53	54	43	0.76
	vic‐raven	56	60	50	0.75
	hmets‐lumped	58	58	57	0.75
	blended‐raven	64	62	59	0.76
	gr4j‐lumped	67	64	69	0.74
	blended‐lumped	68	64	59	0.79
ML	lstm‐lumped	87	81	90	0.82

Further, the overall ranking is consistent with the ordering based on KGE reported in Mai et al. (2022b): the LSTM‐based model is ranked best, followed by PC‐based models that are calibrated per basin, while PC‐based regional models come last. For the regionally calibrated models, there appears to be a linear relationship between overall win percentage and KGE. This relationship breaks down for the better‐performing locally calibrated and ML models. There is one notable difference in the order of locally calibrated models: GR4J is, especially for low‐flow prediction, the best non‐ML‐based model according to expert ratings, but the worst locally calibrated model according to KGE (although only by a small margin).

Appendix A provides a more detailed view on these results, including a paired comparison of models and win percentages broken down by participants' experience and background.

### 3.3 Rating Consistency

In this section, we analyze the quality of responses in terms of their consistency. We build this analysis upon the responses from the second part of our study, where we restricted the number of possible settings to a smaller subset in order to generate duplicate ratings and settings that yield triangle relationships. Accuracy here assesses the frequency where the individual or majority rating agrees with the reference rating.

On average, the ratings from an individual expert have an accuracy of 43% (overall: 37%, high flows: 43%, low flows: 42%) when compared against other ratings of the same setting. This measure improves if we let a panel of experts vote on the rating: the panel of experts as a constructed rater that always gives the majority vote achieves an accuracy of 51% (overall: 48%, high flows: 59%, low flows: 53%). Table 5 provides further details on the performance of individual experts and panels.

**Table 5:** Classification Metrics Averaged Across All Individual Raters When Compared Against Ratings From Other Experts and for the Constructed “Majority Vote” Panel, Where We Compare Each Rating With the Majority Vote of All Other Ratings of the Same Setting (in the Case of a Tie, a Coin Toss Decides)

	Strategy	Model *A* wins	Model *B* wins	Equally good	Equally bad
F1 score	Individual	0.46	0.52	0.12	0.21
	Majority vote	0.60	0.63	0.14	0.31
Precision	Individual	0.51	0.59	0.13	0.27
	Majority vote	0.58	0.63	0.17	0.31
Recall	Individual	0.49	0.55	0.15	0.28
	Majority vote	0.62	0.62	0.12	0.32
Support	Individual^a^	20.35	20.23	5.69	10.42
	Majority vote	122	120	40	63

^a^ These are not integers because the results are averaged across 26 individual raters.

Further, we analyze the consistency of rating triplets that form a triangle relationship of three models. Note that we only consider three rating outcomes for this analysis, which simplifies the definition of consistent triangles (see Section 2.3.2). Our dataset contains 2,507 triangles, out of which we consider 1,658 consistent, that is, 66.13%. This fraction is notably higher than the baseline of random responses, which would yield a consistency of 48.15%.

### 3.4 Metric Ranking

To determine how well the quantitative metrics encode information about the preferences expressed by expert ratings, we trained RF‐based models to predict the ratings of individual samples. Examination of the feature importance indicates how informative each metric is for a certain type of rating. For example, metrics that are more informative regarding the experts' ranking of high flows will be assigned higher degrees of importance in the high‐flow rating task.

**Figure 6:**
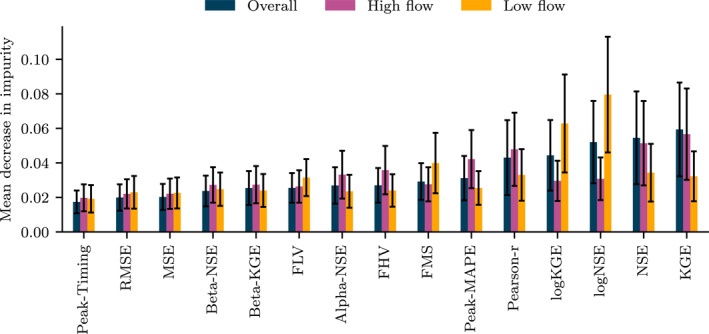
Feature importance from random forests fitted to overall, high‐flow, and low‐flow ratings. Error bars represent the standard deviation across the trees in each forest. The *y*‐axis measures how much a metric contributes toward discriminating the different classes of the training samples; larger values indicate stronger discriminatory power.

The RF‐based models that predict the expert ratings using only the metrics achieve accuracies of around 49%–54%, regardless of the rating task (overall, high flow, low flow). For comparison, recall from Section 3.3 on rating consistency that on average, a human expert achieves accuracies of 37%–43%, and a voting panel achieves 48%–59% (note, however, that the consistency statistics are calculated on the smaller second phase of the study and therefore not fully comparable). Figure 6 indicates the feature (metric) importance for each RF‐based model. For “overall” and “high‐flow” ratings, KGE is selected as the most important metric, while for “low‐flow” ratings, logNSE is selected as most important. Notably, FLV receives comparably small feature importance values for the low flow rating task, despite being explicitly designed for low‐flow evaluation. More generally, the pattern of feature importance obtained for “overall” ratings tends to be similar to that of “high‐flow” ratings, while “low‐flow” ratings follow a different pattern. This aligns with our intuition that hydrologic modelers tend to focus on peaks and high flows rather than seemingly uneventful low flow periods when they are not directed to focus on any specific parts of the hydrographs.

### 3.5 Metric Sufficiency

In Section 3.3, we saw that visual judgment of hydrographs can be very subjective, as indicated by inconsistent ratings that become apparent when comparing multiple ratings of the same settings. Due to these inconsistent outcomes, we cannot expect a set of formal, deterministic metrics to agree with subjective assessments at all times. Yet, our results show that the tested set of quantitative metrics does sufficiently encode information that reflects the patterns that consistently influence expert opinion.

To make this point, we compare the accuracy of two models: first, an RF‐based model that predicts expert ratings purely from the quantitative metrics computed for the two involved models. This classifier does not have direct access to simulated or observed discharge. Second, a GRU‐based model that predicts those ratings directly from the simulated and observed discharge time series data. In theory, this latter model could implicitly calculate any existing or inexistent metric by itself if it helps to predict the ratings. In our experiments, however, the two models achieve approximately the same level of accuracy (RF: 51%, GRU: 50%; see Table 6 for more statistics). Moreover, an accuracy of around 50% is clearly better than the accuracy of individual experts (43%; see Section 3.3. Again, note that the consistency statistics are calculated on the smaller second phase of the study and therefore not fully comparable), and roughly as good as the accuracy of a panel of experts (51%). This indicates that the raw hydrographs do not consistently contain any additional information about the preferences of experts (expressed by their ratings) beyond what is already captured by the metrics.

**Table 6:** Classification Metrics for the Random Forest Purely Based On Metrics and the Gated Recurrent Units Time Series Model Purely Based On Hydrographs

	Decision based on	Model *A* wins	Model *B* wins	Equally good	Equally bad
F1 score	Metrics	0.59	0.61	0.07	0.34
	Hydrographs	0.58	0.61	0.12	0.27
Precision	Metrics	0.53	0.52	0.33	0.44
	Hydrographs	0.52	0.53	0.34	0.38
Recall	Metrics	0.65	0.74	0.04	0.27
	Hydrographs	0.66	0.72	0.07	0.22
Support^a^		816.4	956.4	401.6	628.4

*Note*. The overall accuracy across all four classes is 51% for the random forest and 50% for the GRU. All metrics are averaged across the test results from the five cross‐validation folds.

^a^ These are not integers because the results are averaged across the 5 cross‐validation folds.

## 4 Conclusion

As the first and perhaps least surprising result, this study showed that the superior performance of LSTM‐based streamflow prediction models, as reflected by quantitative metrics, is also reflected in the preferences of humans (when asked to visually rate model performance in a blind study). Given raw hydrograph time series, humans consistently—and by a large margin—prefer the LSTM‐based simulations over those generated by any of the other tested (PC‐based) models.

More importantly, we found that it may not be possible to establish an improved set of quantitative metrics that can provide a more complete representation of human preferences regarding streamflow simulations than what existing metrics provide. Ultimately, this implies that any newly developed metric is likely to either: (a) encode information about model performance that is already captured by (the set of) existing metrics, or (b) encode complementary information that humans do not seem to consider important when evaluating hydrographs. To be clear, neither of these options are necessarily bad. For example, metrics that better encapsulate individual rating patterns (option (a)) or metrics that measure almost imperceptible patterns that are nevertheless important for an application (option (b)) can both be valuable. And, of course, there may also exist new metrics that operate in the space of unpredictable ratings—but the usefulness of a metric that distinguishes patterns that humans cannot agree upon themselves would have to be examined with a great deal of care before being adopted.

We would also like to emphasize the plural in “existing metrics,” since it is clear that no single metric can completely reflect the preferences expressed by the overall rating behavior of humans. This corroborates the general conviction expressed by the community that a single metric does not adequately summarize model performance (e.g., Crochemore et al., 2015; Gupta et al., 1998, 2008, 2009; Legates & McCabe Jr., 1999). Further, the aforementioned apparent sufficiency of existing metrics does not mean that the community should stop working on the development of new metrics. To the contrary, there remains room for the development of new metrics that provide useful information. Our feature importance analysis can serve as a test bench of the alignment between such metrics and human judgment. For example, the RF‐based analysis shows that log‐space metrics (logNSE, logKGE) are especially indicative of human low‐flow ratings, while there was not one individual metric that clearly stood out as the best predictor of high‐flow ratings. In future work, it may even be worthwhile to explore how the time series model that predicts ratings directly from hydrographs could be used as a direct calibration target—although handling the fairly large set of unpredictable ratings may be challenging.

In summary, we interpret these results as an indication that hydrologic practice can, and should, reinforce its use of quantitative metrics to assess streamflow prediction models, while reducing the dependence on subjective visual intuition, except as a form of “sanity check.” While it is unlikely that there will ever be one single metric that is universally useful, our results indicate that the relatively small set of tested metrics (see Table 3) is sufficient to consistently judge the quality of hydrographs. These metrics effectively reduce the high‐dimensional space of time series into a relatively small set of informative numbers. Our results demonstrate that this small set apparently contains all of the information that is deemed to be relevant by human experts when provided with the same task.

## Data Availability

The collected responses as well as the code to generate all results and tables in this manuscript are publicly available at https://doi.org/10.5281/zenodo.7640347 (Gauch et al., 2023) for others to conduct additional analyses in future work. The hydrographs from the GRIP‐GL study are available at https://doi.org/10.20383/103.0598 (see package “A5” for observations and package “A7” for modeled streamflow; Mai et al., 2022a).

## Appendix A Additional Analyses of Model Ranking

Table A1 shows the win percentages across all rating tasks split by each pair of models. In these pairwise comparisons, the best PC‐based contender against the LSTM is Blended‐lumped, but even this model only achieves a win percentage of 25%. The Blended‐lumped model also loses in direct comparison with GR4J, with a win percentage of 40%.

**Table A1:** Win Percentage for Comparisons Between Each Pair of Two Models

	mesh‐class‐raven	gem‐hydro‐watroute	mesh‐svs‐raven	swat‐raven	watflood‐raven	lbrm‐cc‐lumped	hymod2‐lumped	vic‐raven	hmets‐lumped	blended‐raven	gr4j‐lumped	blended‐lumped	lstm‐lumped
mesh‐class‐raven		50	31	35	37	20	23	22	22	22	16	21	7
gem‐hydro‐watroute	50		35	43	35	21	33	25	18	24	16	16	5
mesh‐svs‐raven	69	65		49	50	45	32	29	31	26	21	32	14
swat‐raven	65	57	51		47	28	31	30	29	24	19	23	7
watflood‐raven	63	65	50	53		33	34	34	26	29	17	25	15
lbrm‐cc‐lumped	80	79	55	72	67		34	42	38	31	37	35	12
hymod2‐lumped	77	67	68	69	66	66		38	41	29	31	36	14
vic‐raven	78	75	71	70	66	58	62		55	41	35	37	16
hmets‐lumped	78	82	69	71	74	62	59	45		49	42	46	14
blended‐raven	78	76	74	76	71	69	71	59	51		48	40	19
gr4j‐lumped	84	84	79	81	83	63	69	65	58	52		60	18
blended‐lumped	79	84	68	77	75	65	64	63	54	60	40		25
lstm‐lumped	93	95	86	93	85	88	86	84	86	81	82	75	

*Note*. The value in row *i*, column *j* denotes the win percentage of model *i* when compared against model *j*.

Because we collected demographic information from our participants, we can break down the ratings by demographic groups. Tables A2 and A3 differentiate the win percentages of each model by participant experience and employment sector, respectively. Ratings differ significantly across these groups. In particular, participants with between 10 and 20 years of experience and those who work in industry chose the LSTM‐based model as being better much more often than those with fewer experience or those in academia.

**Table A2:** Win Percentage by Model, Split by Participants' Experience

	Years of experience	Win percentage				
		<5	5–10	10–15	15–20	≥20
	Num. of participants	168	151	121	67	115
Regionally calibrated	mesh‐class‐raven	22	28	28	21	21
	gem‐hydro‐watroute	24	25	26	25	27
	mesh‐svs‐raven	36	35	31	39	42
	swat‐raven	33	38	30	34	30
	watflood‐raven	35	35	32	34	39
Locally calibrated	lbrm‐cc‐lumped	46	49	43	52	52
	hymod2‐lumped	51	50	51	48	49
	vic‐raven	53	56	56	55	57
	hmets‐lumped	55	58	59	55	61
	blended‐raven	64	62	69	61	57
	gr4j‐lumped	70	63	68	61	69
	blended‐lumped	66	65	62	68	60
ML	lstm‐lumped	86	83	90	90	87

**Table A3:** Win Percentage by Model, Split by Participants' Sector

	Group	Win percentage		
		Academia	Public sector	Industry
	Num. of participants	408	122	92
Regionally calibrated	mesh‐class‐raven	25	23	20
	gem‐hydro‐watroute	26	23	29
	mesh‐svs‐raven	35	41	40
	swat‐raven	32	33	38
	watflood‐raven	37	34	29
Locally calibrated	lbrm‐cc‐lumped	47	50	51
	hymod2‐lumped	49	52	54
	vic‐raven	57	54	50
	hmets‐lumped	58	56	61
	blended‐raven	62	63	61
	gr4j‐lumped	68	65	64
	blended‐lumped	65	64	61
ML	lstm‐lumped	85	88	91

Moreover, participants whose work focuses on drought forecasting rate the models differently than most other participants (Table A4). To them, the PC‐based LBRM and VIC models are best for high flows, while the LSTM‐based model ranks only third. Additionally, drought modelers were much less clear in their preference of the LSTM for low‐flow predictions. Whereas flood modelers picked the LSTM‐based model for low flows with a win percentage of 88%, drought modelers did so only with 81%—still selecting it as the best model, but not quite as clearly. As the second‐favorite of drought modelers for low flows, GR4J achieves a win percentage of 77%.

**Table A4:** Win Percentage by Model, Split by Rating Task and Whether Participants Focus on Flood or Drought Modeling

	Focus area	Win percentage					
		Flood but not drought			Drought but not flood		
	Rating task	Overall	High fl.	Low fl.	Overall	High fl.	Low fl.
	Num. participants	253	223	212	38	33	30
Regionally calibrated	mesh‐class‐raven	24	33	22	25	59	26
	gem‐hydro‐watroute	21	23	30	26	21	45
	mesh‐svs‐raven	28	34	54	33	38	45
	swat‐raven	35	32	31	37	37	40
	watflood‐raven	40	34	32	42	52	35
Locally calibrated	lbrm‐cc‐lumped	49	48	46	37	65	27
	hymod2‐lumped	57	57	42	53	50	43
	vic‐raven	55	59	52	51	63	52
	hmets‐lumped	57	57	59	73	36	44
	blended‐raven	65	60	61	62	52	58
	gr4j‐lumped	66	70	68	69	60	77
	blended‐lumped	65	62	59	58	56	70
ML	lstm‐lumped	83	83	88	80	61	81

## Appendix B Classification Metrics

This section introduces the classification metrics accuracy, precision, recall, and F1 score in the context of our study. Originally, these concepts stem from the information retrieval community (Klampanos, 2009), but they have proven useful in the evaluation of various other classification problems. For the following definitions, we use the following notation: Let $\left\{{\hat{r}}_{1}^{j},\text{…},{\hat{r}}_{n_{j}}^{j}\right\}$ be the set of ratings for setting *j* ∈ {1, …, *m*}, each representing one rater's opinion ${\hat{r}}_{i}^{j}\in C=\left\{\text{model}\;A,\text{model}\;B,\text{equally}\;\text{good},\text{equally}\;\text{bad}\right\}$. Further, let $r^{j}\in C$ be the corresponding “correct” judgment for some definition of correctness (e.g., an arbitrarily selected hold‐out rating or a majority vote, see Section 2.3.2). We call *r* ^*j*^ the *ground truth* for ratings of setting *j*.

Given these ratings and ground truth values, we can define the number of true and false positives (TP_*c*_, FP_*c*_) as well as of true and false negatives (TN_*c*_, FN_*c*_) for each class c∈C:

(B1)
$${\text{TP}}_{c}=|\left\{{\hat{r}}_{i}^{j}|{\hat{r}}_{i}^{j}=r^{j}=c\right\}|$$

(B2)
$${\text{FP}}_{c}=|\left\{{\hat{r}}_{i}^{j}|{\hat{r}}_{i}^{j}=c\neq r^{j}\right\}|$$

(B3)
$${\text{TN}}_{c}=|\left\{{\hat{r}}_{i}^{j}|{\hat{r}}_{i}^{j}\neq c\;\text{and}\;r^{j}\neq c\right\}|$$

(B4)
$${\text{FN}}_{c}=|\left\{{\hat{r}}_{i}^{j}|{\hat{r}}_{i}^{j}\neq r^{j}=c\right\}|.$$

**Accuracy** is defined as the number of correct judgments, divided by the total number of predictions:

(B5)
$$\text{Acc}=\frac{\sum_{c\in C}{\text{TP}}_{c}}{\sum_{c\in C}\left({\text{TP}}_{c}+{\text{FP}}_{c}\right)}$$

**Precision** is defined for each possible class individually. For class *c*, the precision measures how many of the ratings that decided for *c* were in agreement with the corresponding correct rating:

(B6)
$${\text{Pr}}_{c}=\frac{{\text{TP}}_{c}}{{\text{TP}}_{c}+{\text{FP}}_{c}}.$$

**Recall** describes how many of the settings that, according to the ground truth, belong to class *c* were actually labeled as *c* by the raters:

(B7)
$${\text{Re}}_{c}=\frac{{\text{TP}}_{c}}{{\text{TP}}_{c}+{\text{FN}}_{c}}.$$

Finally, the **F1 score** is the is the harmonic mean of precision and recall:

(B8)
$${F1}_{c}=2\;\frac{{\text{Pr}}_{c}\cdot {\text{Re}}_{c}}{{\text{Pr}}_{c}+{\text{Re}}_{c}}.$$
